# Aβ_42_-mediated proteasome inhibition and associated tau pathology in hippocampus are governed by a lysosomal response involving cathepsin B: Evidence for protective crosstalk between protein clearance pathways

**DOI:** 10.1371/journal.pone.0182895

**Published:** 2017-08-10

**Authors:** Karen L. G. Farizatto, Uzoma S. Ikonne, Michael F. Almeida, Merari F. R. Ferrari, Ben A. Bahr

**Affiliations:** 1 Biotechnology Research and Training Center, William C. Friday Laboratory, University of North Carolina—Pembroke, Pembroke, North Carolina, United States of America; 2 Department of Genetics and Evolutionary Biology, Institute for Biosciences, University of Sao Paulo, Sao Paulo, Sao Paulo, Brazil; USF Health Morsani College of Medicine, UNITED STATES

## Abstract

Impaired protein clearance likely increases the risk of protein accumulation disorders including Alzheimer’s disease (AD). Protein degradation through the proteasome pathway decreases with age and in AD brains, and the Aβ_42_ peptide has been shown to impair proteasome function in cultured cells and in a cell-free model. Here, Aβ_42_ was studied in brain tissue to measure changes in protein clearance pathways and related secondary pathology. Oligomerized Aβ_42_ (0.5–1.5 μM) reduced proteasome activity by 62% in hippocampal slice cultures over a 4-6-day period, corresponding with increased tau phosphorylation and reduced synaptophysin levels. Interestingly, the decrease in proteasome activity was associated with a delayed inverse effect, >2-fold increase, regarding lysosomal cathepsin B (CatB) activity. The CatB enhancement did not correspond with the Aβ_42_-mediated phospho-tau alterations since the latter occurred prior to the CatB response. Hippocampal slices treated with the proteasome inhibitor lactacystin also exhibited an inverse effect on CatB activity with respect to diminished proteasome function. Lactacystin caused earlier CatB enhancement than Aβ_42_, and no correspondence was evident between up-regulated CatB levels and the delayed synaptic pathology indicated by the loss of pre- and postsynaptic markers. Contrasting the inverse effects on the proteasomal and lysosomal pathways by Aβ_42_ and lactacystin, such were not found when CatB activity was up-regulated two-fold with Z-Phe-Ala-diazomethylketone (PADK). Instead of an inverse decline, proteasome function was increased marginally in PADK-treated hippocampal slices. Unexpectedly, the proteasomal augmentation was significantly pronounced in Aβ_42_-compromised slices, while absent in lactacystin-treated tissue, resulting in >2-fold improvement for nearly complete recovery of proteasome function by the CatB-enhancing compound. The PADK treatment also reduced Aβ_42_-mediated tau phosphorylation and synaptic marker declines, corresponding with the positive modulation of both proteasome activity and the lysosomal CatB enzyme. These findings indicate that proteasomal stress contributes to AD-type pathogenesis and that governing such pathology occurs through crosstalk between the two protein clearance pathways.

## Introduction

Degradation of old and damaged proteins, through the proteasome and autophagy-lysosome systems, decreases with age thus altering the vital balance between protein synthesis and protein clearance (see reviews: [1–4]). This altered balance influences age-related neurodegenerative disorders, likely increasing the risk of protein accumulation disorders including Alzheimer’s disease (AD) since deposition of amyloid and tau proteins develop long before the onset of cognitive symptoms [5]. Altered levels of lysosomes, impaired maturation of autophagolysosomes, and other stress indicators of the autophagy-lysosomal pathway occur in AD brains [6–8]. AD and mild cognitive impairment also exhibit compromised proteasome function as do other age-related diseases [9–13]. Proteasomal dysfunction in the AD brain has been shown to result from the inhibitory binding of filamentous tau to proteasomes [14], and a recent study found that tau-driven proteasome impairment occurs in a mouse model of tauopathy [15]. In addition, Aβ_42_ peptide oligomers inhibit proteasomes *in vitro* [16, 17], and 3xTg-AD mice exhibit impaired proteasome activity correlating with intraneuronal oligomers [16]. The study also found that proteasome inhibitors lead to increases in Aβ and tau levels in pre-pathological 3xTg-AD mice, thus pointing to proteasomal stress as a contributor to the multi-proteinopathy of AD.

The proteasome and autophagy-lysosomal systems do not appear to be completely independent protein clearance pathways [18–21], but rather exhibit evidence of concerted regulation and crosstalk to maintain protein homeostasis. As an example of such crosstalk, autophagy upregulation has been implicated as a response to compensate for proteasomal stress [17, 22–24], with autophagy activation and lysosomal recruitment occurring in an HDAC6-dependent manner. Also, proteasome inhibition has been shown to activate autophagy in order to eliminate protein aggregates in human cancer cells and mouse fibroblasts [25], and such activation of the lysosomal proteolytic pathway has been linked to chronic, low-level proteasome inhibition in neural SH-SY5Y cells [26]. Early activation of the autophagic-lysosomal pathway may explain the abnormally high number of lysosomal compartments containing cathepsin B (CatB) within neurons of at-risk regions from sporadic AD brains [27]. A positive modulator of the autophagy-lysosomal system was recently found to enhance CatB activity and to reduce Aβ_42_ accumulation in neuroblastoma cells expressing human amyloid precursor protein (APP) with the Swedish mutation [28]. It is noteworthy that CatB regulation has been extensively studied and the enzyme was found to be increased in response to the following types of protein accumulation stress:

- chloroquine-induced protein accumulation stress in hippocampal slices [29]
- human huntingtin expression in cultured neurons [30]
- Aβ_42_ treatment of mouse neuronal cell line [31]
- expression of familial AD mutant APP in transgenic mouse brain [31]
- proteasome inhibitor treatment in rat hippocampus [32]
- proteasome inhibitor treatment in control SH-SY5Y cells and those expressing human APP [33]

The above CatB responses are of interest since such cellular responses may link the proteasomal and lysosomal pathways during episodes of protein accumulation pathology. The responses may in fact influence AD pathogenesis since CatB degrades Aβ_42_ through C-terminal truncation [31, 34, 35], and such degradation was blocked by the selective CatB inhibitor CA074 [31]. A similar inhibitor blocked the ability of a CatB-enhancing compound from improving Aβ_42_ clearance in monocytes from AD patients [36]. In several transgenic mouse models of AD, genetic and pharmacological treatments that increase CatB activity led to the significant lowering of Aβ_42_ levels [31, 35, 37–41] as well as to reductions in the cellular and behavioral disease parameters [31, 35, 37, 38, 41]. However, there is conflicting evidence about the involvement of CatB in regards to protein accumulation events since reducing CatB has also been shown to lower Aβ peptide levels and improve memory in transgenic mice [42, 43]. Due to the conflicting reports, it is important to further address the role CatB plays during protein accumulation pathology. Many questions obviously remain regarding the CatB enzyme and the different roles proposed for it in the brain.

Protein clearance is vital for cellular homeostasis and the clearance rates for Aβ peptides are indeed slower in AD as compared to cognitively normal individuals [44]. Accordingly, we set out to understand how Aβ_42_ and a selective proteasome inhibitor influence the interplay between the proteasomal and lysosomal protein clearance pathways in brain tissue. Using cultured slices of a brain area that is vulnerable to AD, the present study will also examine CatB’s response to proteasomal stress in order to understand its role in secondary pathogenic events.

## Materials and methods

### Hippocampal slice cultures

Sprague-Dawley rat litters (Charles River Laboratories, Wilmington, MA) were housed and treated in accordance with the recommendations from the Guide for the Care and Use of Laboratory Animals from the National Institutes of Health and in accordance with an approved protocol from the Institutional Animal Care and Use Committee of the University of North Carolina—Pembroke. Brain tissue from postnatal 12-day-old rats was rapidly removed to prepare hippocampal slices [29, 45]. Transverse slices of hippocampus (400 μm) were quickly prepared and the slices were kept in an iced buffer solution until groups of slices could be gently positioned on the Biopore PTFE membrane of each Millicell-CM insert (Millipore, Billerica, MA). Culture medium consisting of 50% basal medium Eagle, 25% Earle's balanced salts, 25% horse serum (Gemini Bio-products, Sacramento, CA), and defined supplements as described [45] was changed periodically. The hippocampal slices were maintained at 37°C in 5% CO_2_-enriched atmosphere for 18–22 days before being treated with different agents. A subset of slices were fixed and subjected to anti-synaptophysin immunocytochemistry, and images were acquired using Zeiss 710 and Zeiss 780 confocal systems.

### Treatments

Aβ_42_ peptide (American Peptide Company, Sunnyvale, CA) from precise aliquots was reconstituted in DMSO at 20 mM and diluted to 15 μM in phosphate-buffered saline. The diluted peptide was allowed to aggregate in the dark at room temperature for 6 days before being utilized for experiments in hippocampal slices cultures. Over a period of 4–6 days, pre-aggregated Aβ_42_ diluted to 0.5–1.5 μM in serum-free media was applied to the cultured slices for 14–16 h. Subsequently, horse serum-containing medium was mixed into culture wells, further diluting Aβ_42_ to 0.38–1.1 μM for 8–10 h before the next daily treatment. In other treatments, the irreversible proteasome inhibitor lactacystin (Santa Cruz Biotechnology, Santa Cruz, CA) was applied daily to hippocampal slice cultures at 5–10 μM for 1–4 days. Other compounds and lysosome-targeting agents (see ref. [46]) were also tested in the hippocampal slices to identify CatB positive modulators. The compound Z-Phe-Ala-diazomethylketone (PADK, also known as Z-FA-CHN_2_; Bachem, King of Prussia, PA) that promotes mature CatB levels in different model systems [35, 47, 48] was diluted to 1–10 μM and applied daily to cultures in the absence or presence of Aβ_42_. After the treatments, cultured slices were fixed in 4% paraformaldehyde for histology or gently removed from the inserts into groups of 7–9 each using ice-cold isosmotic buffer containing 0.32 M sucrose, 5 mM HEPES (pH 7.4), 1 mM EDTA and 1 mM EGTA.

### Immunoblot analysis

The groups of hippocampal slices were centrifuged and the pelleted tissue was sonicated in cold lysis buffer consisting of 15 mM HEPES (pH 7.4), 0.5 mM EDTA, 0.5 mM EGTA, and a protease inhibitor cocktail (Sigma-Aldrich; St. Louis, MO). Protein content was determined using the Pierce BCA Protein Assay (Thermo Scientific, Rockford, IL). Equal protein aliquots of the hippocampal slice samples were denatured in sample buffer for 5 min at 100°C and the proteins separated on gradient gels and transferred to nitrocellulose. Blots were incubated in blocking solution containing 5% milk or BSA for 1 h. Primary antibody staining utilized antibodies against synaptophysin (1:1000), synapsin II (1:500), cathepsin B (1:100), and GluR1 (1:500) from Millipore, as well as against actin (1:500) and phospho-tau^Ser199/202^ (1:1000) from Sigma-Aldrich and 20S proteasome α-1 subunit (1:500) from Santa Cruz Biotechnology. Anti-IgG-alkaline phosphatase conjugates and anti-IgG-horseradish peroxidase conjugates were used for the secondary antibody step, and antigen staining and image development involved the 5-bromo-4-chloro-3-indolyl phosphate and nitroblue tetrazolium substrate system or chemiluminescence protocols using Hyperfilm ECL exposures (GE Healthcare, Pittsburgh, PA) as well as the GE Amersham AI600RGB imager. Immunostained bands were scanned at high resolution to determine integrated optical density with BIOQUANT software (R & M Biometrics, Nashville, TN).

### Proteasome activity

The suc-LLVY-AMC substrate was used to assess the chymotrypsin-like proteasome activity in hippocampal slice cultures. Samples of freshly harvested slice tissue were quickly homogenized in assay buffer and equal protein aliquots (30 μg) in duplicate were incubated with a fluorogenic substrate (Millipore) in 20 mM Tris buffer, pH 7.5, at 37°C. The fluorescence emission at 440 nm was measured periodically over a 45-min period using the SpectraMax M3 microplate reader. Lactacystin was added to a subset of control slice samples to determine that >80% of the measured substrate cleavage was mediated by lactacystin-sensitive proteasome activity.

### Cathepsin B activity

The InnoZyme Cathepsin B Activity Assay Kit (Millipore) was used to measure CatB activity in the hippocampal slice samples. Aliquots of homogenized samples (10 μg protein) were assessed in duplicate for proteolytic activity using the Z-Arg-Arg AMC substrate and the SpectraMax M3 microplate reader. The CatB specific inhibitor CA074 (10 μM) was added to a subset of control slice samples to determine that all of the measured substrate cleavage was mediated by CatB.

### Statistical analyses

For the results of mean and SEM values across treatment groups, statistical tests included linear regression analysis, unpaired *t* tests, and analyses of variance (ANOVA) followed by post hoc tests using Prism software (GraphPad, San Diego, CA).

## Results

**Fig 1:**
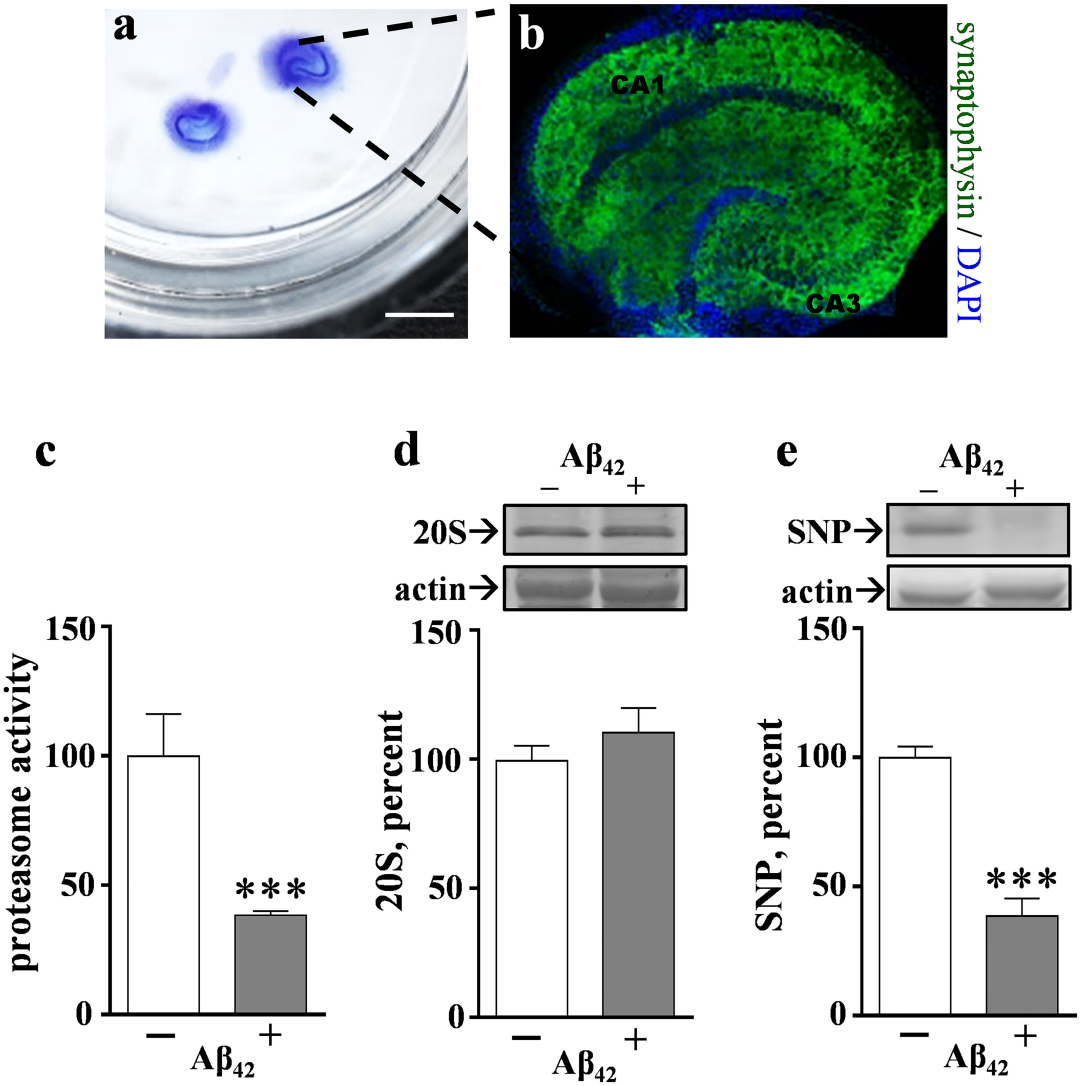
Pre-aggregated Aβ_42_ causes proteasomal dysfunction in correspondence with synaptic decline in rat hippocampal slice cultures. The brain tissue was maintained on culture inserts with media placed below the insert membrane—note the two Nissl-stained slices showing their insert positions and neuronal layers (a) (size bar = 3 mm). After several weeks in culture, a hippocampal slice was stained with anti-synaptophysin and DAPI to show the stable maintenance of CA1, CA3, and dentate gyrus subfields and their associated dense neuropil (b) (view-field width: 2.6 mm). Aliquots of pre-aggregated, human Aβ_42_ peptide were diluted to 0.5–1.5 μM and applied daily to slice cultures alongside vehicle-treated slices. Proteasome chymotrypsin-like activity (mean Vmax/s ± SEM normalized to control; n = 6) was measured in hippocampal slices that were harvested after 4–6 days of treatment (c). For immunoblots, groups of 7–9 slices each were harvested after 6 days of treatment, sonicated, and equal protein aliquots assessed for the 20S proteasome α-1 subunit (d), synaptophysin (SNP; e), and actin. Mean immunoreactivity levels were normalized to their respective controls and percent ± SEM values are shown. Unpaired t-tests: ***p< 0.001.

To test for changes in the proteasome pathway that are related to the AD Aβ_42_ peptide, the peptide’s effects were assessed in stable explants of brain tissue. Rat hippocampal slice cultures were used, a method providing mature brain tissue that maintains native neuronal organization and synaptic density (Fig 1a and 1b) as well as other features of the adult hippocampus [49]. Aβ_42_ was first allowed to self-aggregate for 6 days following a previously established protocol [50] in order to evaluate oligomeric species thought to be involved in AD-related stress on cellular homeostasis. Hippocampal slice cultures were treated daily with the pre-aggregated, low-concentration Aβ_42_ and as a result proteasome chymotrypsin-like activity was reduced by more than 60% after 4–6 days (Fig 1c). Note that the proteasome assay measured activity that is sensitive to 20S proteasome inhibitors, but the loss of proteasome function due to Aβ_42_ occurred in the absence of any change in the 20S proteasome α-1 subunit (Fig 1d).

**Fig 2:**
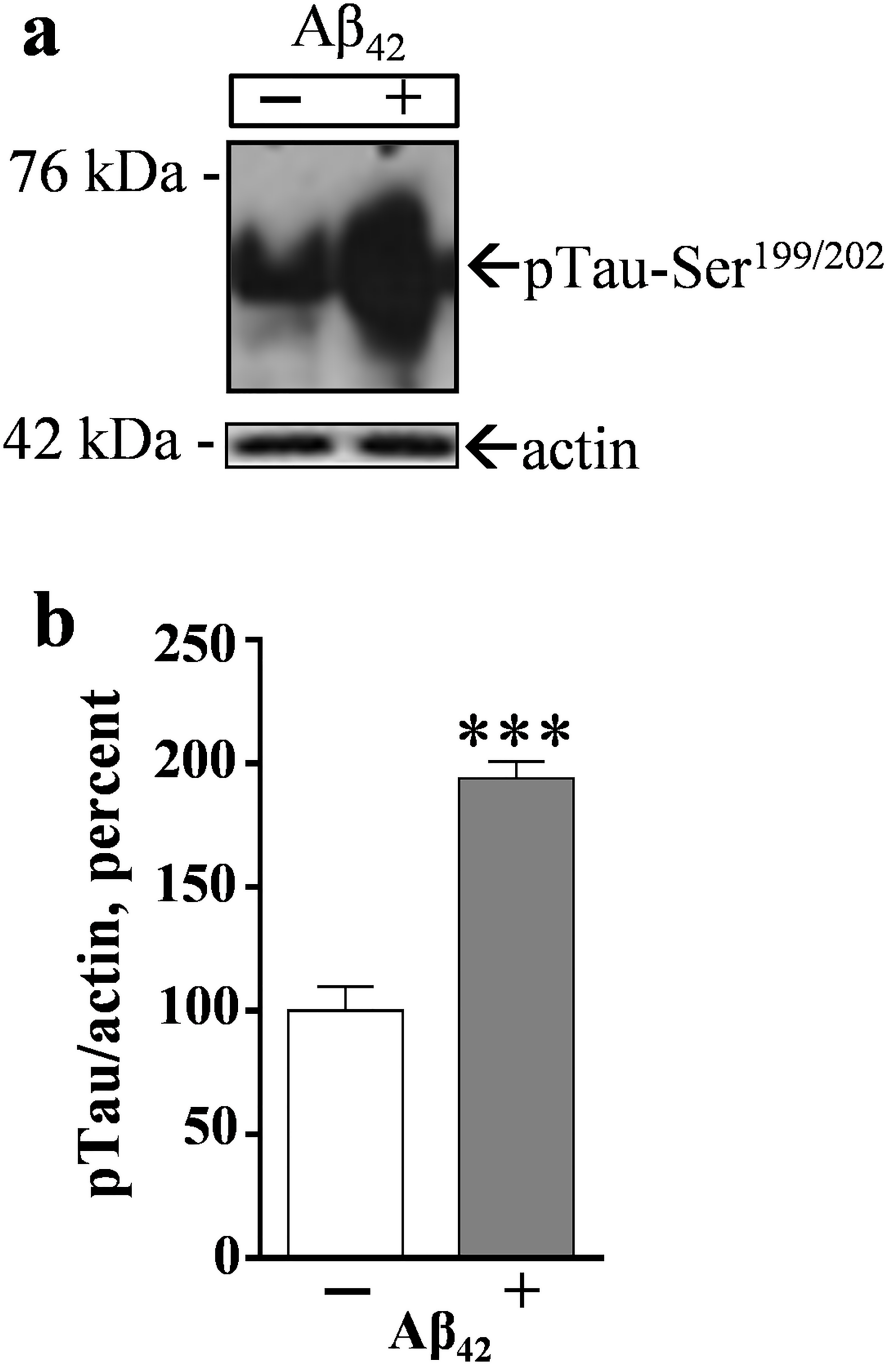
Treatment with pre-aggregated Aβ_42_ peptide leads to increased phosphorylation of tau residues Ser199 and Ser202. Hippocampal slice cultures were treated daily with Aβ_42_ alongside vehicle-treated slices. After 6 days the tissue was gently harvested into groups of 7–9 slices each and equal protein aliquots assessed by immunoblot with antibodies against phospho-tau-Ser^199/202^ and against actin (a). Positions of molecular weight standards are shown. The 55–70-kDa phospho-tau immunoreactivity levels were normalized to within-sample actin measures and plotted as mean percent of control ± SEM (b). Unpaired t-test: ***p< 0.001.

The compromised proteasome activity was tested for a link to secondary synaptic pathology since the proteasome pathway has a major role in regulating synaptic proteins [51]. The Aβ_42_-induced proteasome inhibition was indeed associated with a significant loss in synaptophysin (Fig 1e). The presynaptic marker was reduced by 61% as compared to control hippocampal slice cultures, whereas other proteins including actin were unchanged. Note that the reductions in synaptophysin and proteasome activity were similar in degree and they occurred in correspondence with an Aβ_42_-mediated increase in tau species, isoforms identified as being phosphorylated on Ser199 and Ser202 (Fig 2). Thus, the effects of proteasome dysfunction in the brain may involve a previously identified pathogenic cascade linking tau deposition and gradual synaptic decline [45]. The 55- to 70-kDa phospho-tau immunoreactivity, normalized to within-sample actin levels, was increased two-fold after hippocampal slices were treated for 6 days with 0.5–1.5 μM pre-aggregated Aβ_42_ (p<0.0001).

**Fig 3:**
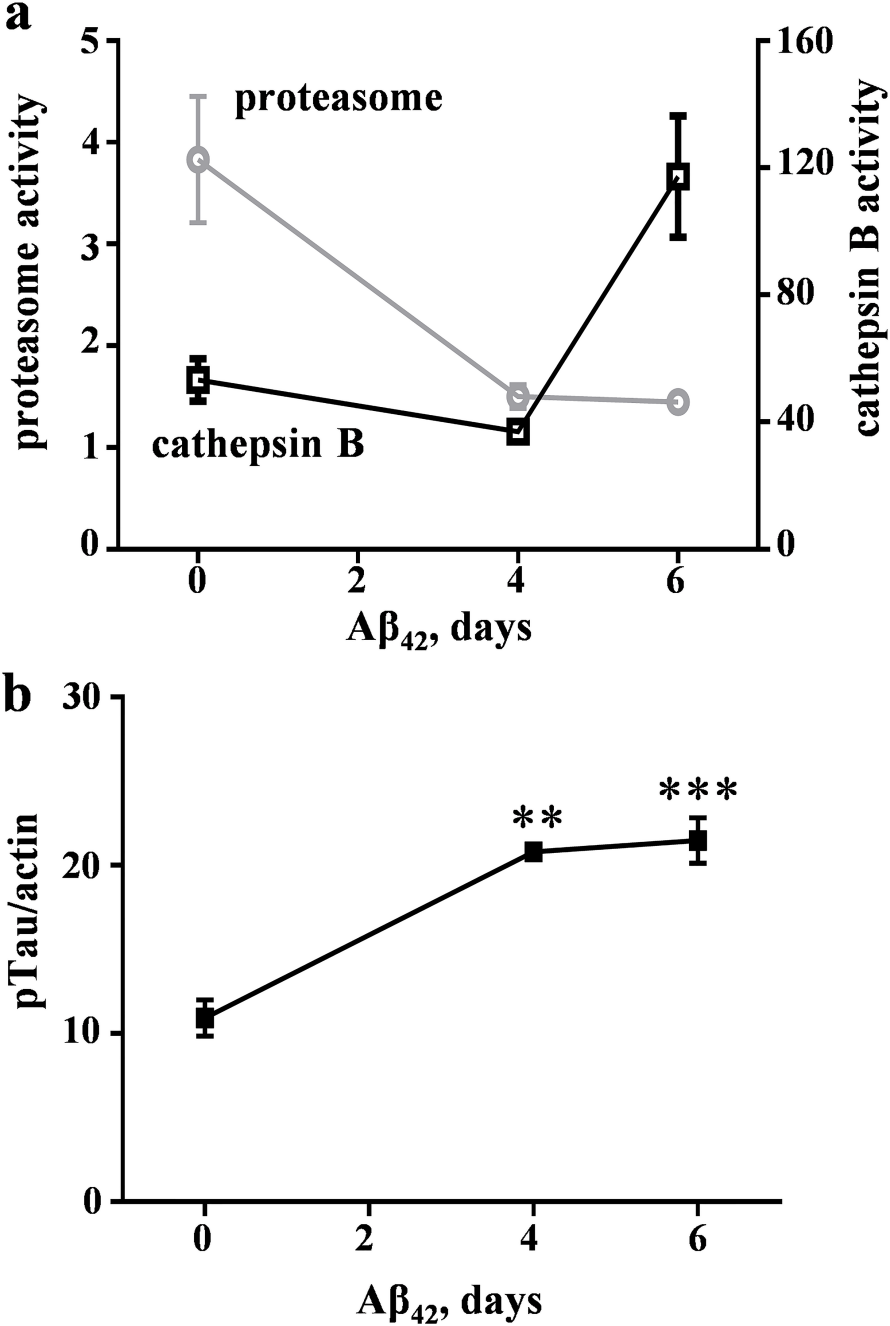
Aβ_42_-induced proteasome inhibition is associated with a delayed, inverse effect on CatB activity. Hippocampal slice cultures were treated daily with vehicle for 6 days (0-day control group) or with pre-aggregated Aβ_42_ for 4–6 days, staggering the treatments in order for same-day harvesting of slice groups of 7–9 each. Proteasome activity (gray plot of mean Vmax/s ± SEM; ANOVA: p<0.01) and cathepsin B activity (black plot of mean fluorometric units ± SEM; ANOVA: p<0.01) were measured in equal protein aliquots from the same samples (a). The time-course samples were also assessed for 55–70-kDa pTau-Ser^199/202^ and actin in order to plot the within-sample ratios between the immunoreactivity levels (b; mean ± SEM). Tukey post hoc tests compared to vehicle-treated slices: **p<0.01, ***p<0.001.

Next, we tested whether the Aβ_42_-mediated proteasome compromise leads to changes in the lysosomal pathway since Aβ_42_ preparations [29, 31, 52] and proteasome inhibitors [32, 33] have been shown to increase lysosomal cathepsin enzymes. In hippocampal slice cultures, proteasome activity was significantly decreased 62% by a preparation of pre-aggregated Aβ_42_ over a 6-day treatment period (Fig 3a). Note after 4 days of Aβ_42_ treatment, the reduction in proteasome function was not associated with any activity change regarding the lysosomal enzyme CatB. However, it is of interest that after 6 days of treatment the CatB activity exhibited a striking 2–3-fold increase over the baseline level (p = 0.0025). The delayed up-regulation in CatB activity shown in Fig 3a does not correspond with Aβ_42_-mediated increases in pTau-Ser^199/202^ species (Fig 3b) since the latter occurred prior to the CatB response.

**Fig 4:**
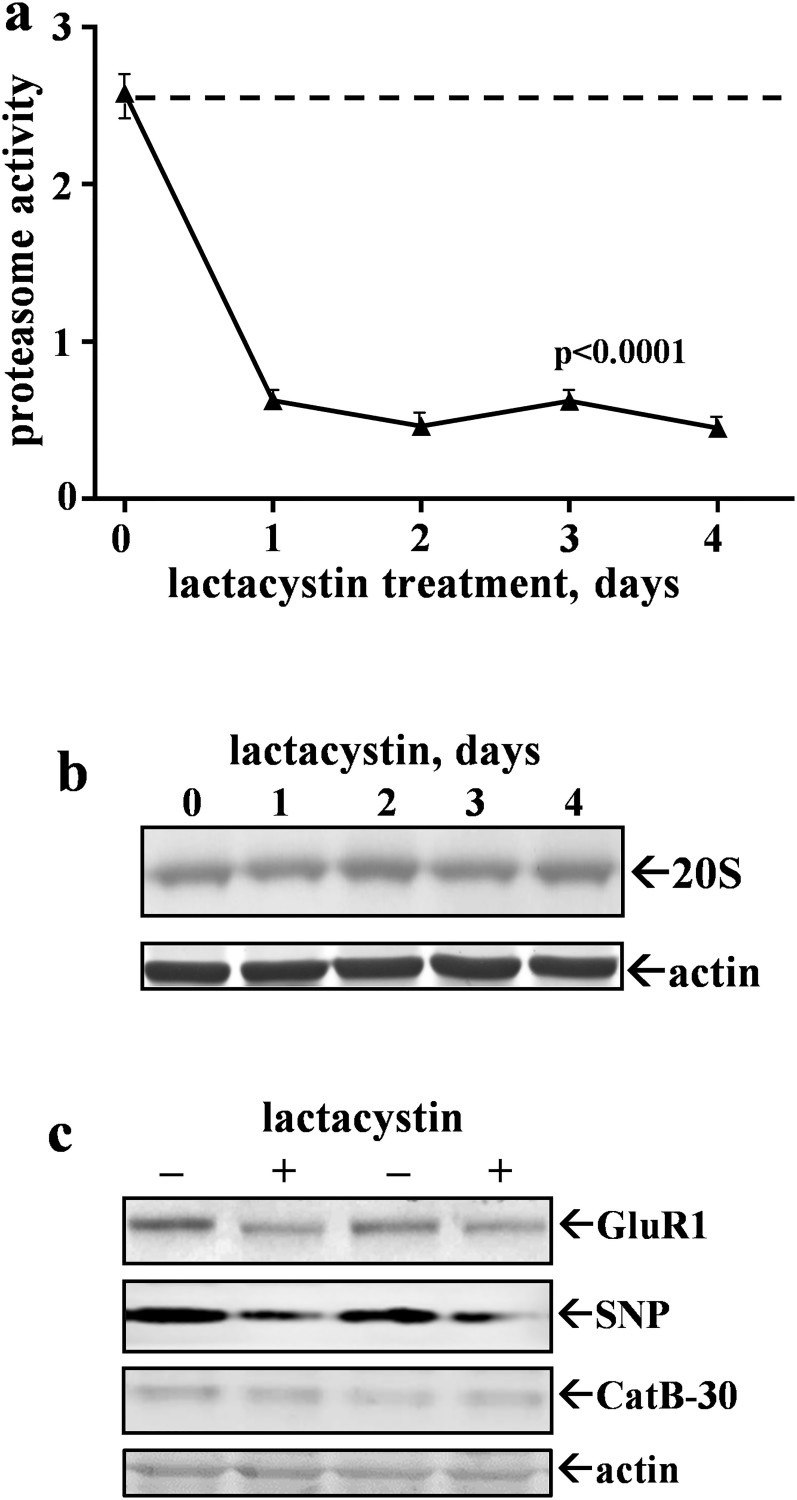
Proteasome activity is blocked by the inhibitor lactacystin in hippocampal slice cultures. The slices were treated daily with vehicle for 4 days (0-day control group) or with 5 μM lactacystin for 1–4 days, staggering the treatments in order for same-day preparation of slice groups of 7–9 each. Proteasome activity (mean Vmax/s ± SEM) was measured in control slices harvested at different times (dashed line) and in lactacystin-treated slice samples (a). The time-course data were analyzed by ANOVA (p<0.0001; post hoc tests compared to control: p<0.0001 at all 4 time points). A subset of the samples tested for proteasome activity was also assessed by immunoblot for the 20S proteasome α-1 subunit (20S) and actin (b), as well as for GluR1, synaptophysin (SNP), the 30-kDa CatB isoform (CatB-30), and again the actin load control (c).

**Fig 5:**
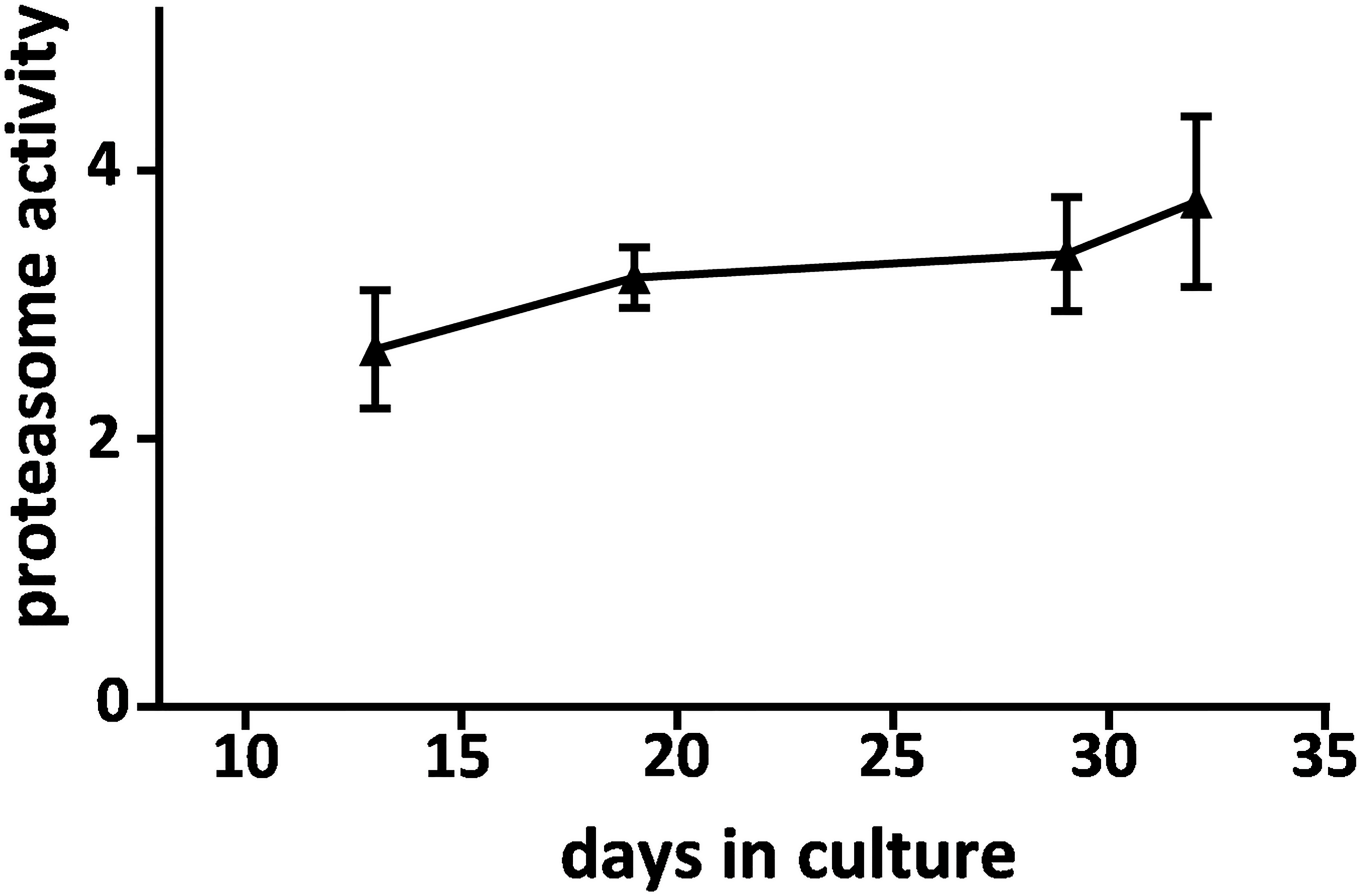
Hippocampal slice cultures exhibit stable proteasomal activity. **Proteasome activity was measured with a fluorogenic peptide assay in control hippocampal slices harvested across a wide range of culture days**. The mean Vmax/s ± SEM data were plotted across days in culture for the different slice samples in order to verify stable maintenance of proteasome function in the tissue model.

To test if CatB responds to another type of proteasomal stress, cultured hippocampal slices were treated with the 20S proteasome inhibitor lactacystin. As with Aβ_42_ treatment, lactacystin significantly reduced proteasome activity (Fig 4a) but it had a larger inhibitory effect than that produced by the pre-aggregated Aβ_42_. The dashed line in Fig 4a represents control proteasome activity, and Fig 5 confirms that mature hippocampal slice cultures stably maintain proteasomal activity across several weeks in culture. The lactacystin-induced decline in proteasome function was also found to be independent of any change in the 20S proteasome α-1 subunit (Fig 4b) as was found with Aβ_42_. Comparing lactacystin with Aβ_42_ with regards to CatB modulation, lactacystin had an inverse effect on CatB activity, as did Aβ_42_, with respect to the inhibitory action on the proteasome pathway (Table 1). A difference was apparent in that lactacystin elicited earlier CatB up-regulation compared to Aβ_42_’s effect that was delayed more than 4 days. A 28% increase in CatB activity was found after only the first day of lactacystin treatment (nearing significance with p = 0.054), and subsequent treatment days resulted in markedly up-regulated activity, increasing by 62–109% in a time-dependent manner (p<0.001). Note that the increase in CatB activity by lactacystin was not associated with any change in the 30-kDa active isoform of the CatB enzyme (see Fig 4c). The 30-kDa isoform in slice samples with 2–4 days of treatment was 117 ± 20.3% of control (n = 7; N.S.).

**Table 1:** Lactacystin-induced proteasome inhibition leads to the enhancement of CatB activity.

Treatment	days	CatB activity, percent ± SEM
vehicle	4	100 ± 10.7
lactacystin	1	128 ± 8.7
lactacystin	2	162 ± 10.2*
lactacystin	3	209 ± 20.2***
lactacystin	4	204 ± 4.6***

Hippocampal slice cultures were treated daily with vehicle for 4 days or with 5 μM lactacystin for 1–4 days, staggering the treatments in order for same-day preparation of slice groups of 7–9 each. CatB activity was measured in equal protein aliquots of samples and fluorometric units were normalized to vehicle-treated control samples. Tukey post hoc tests compared to vehicle treatment:

*p = 0.028.

***p<0.001.

**Fig 6:**
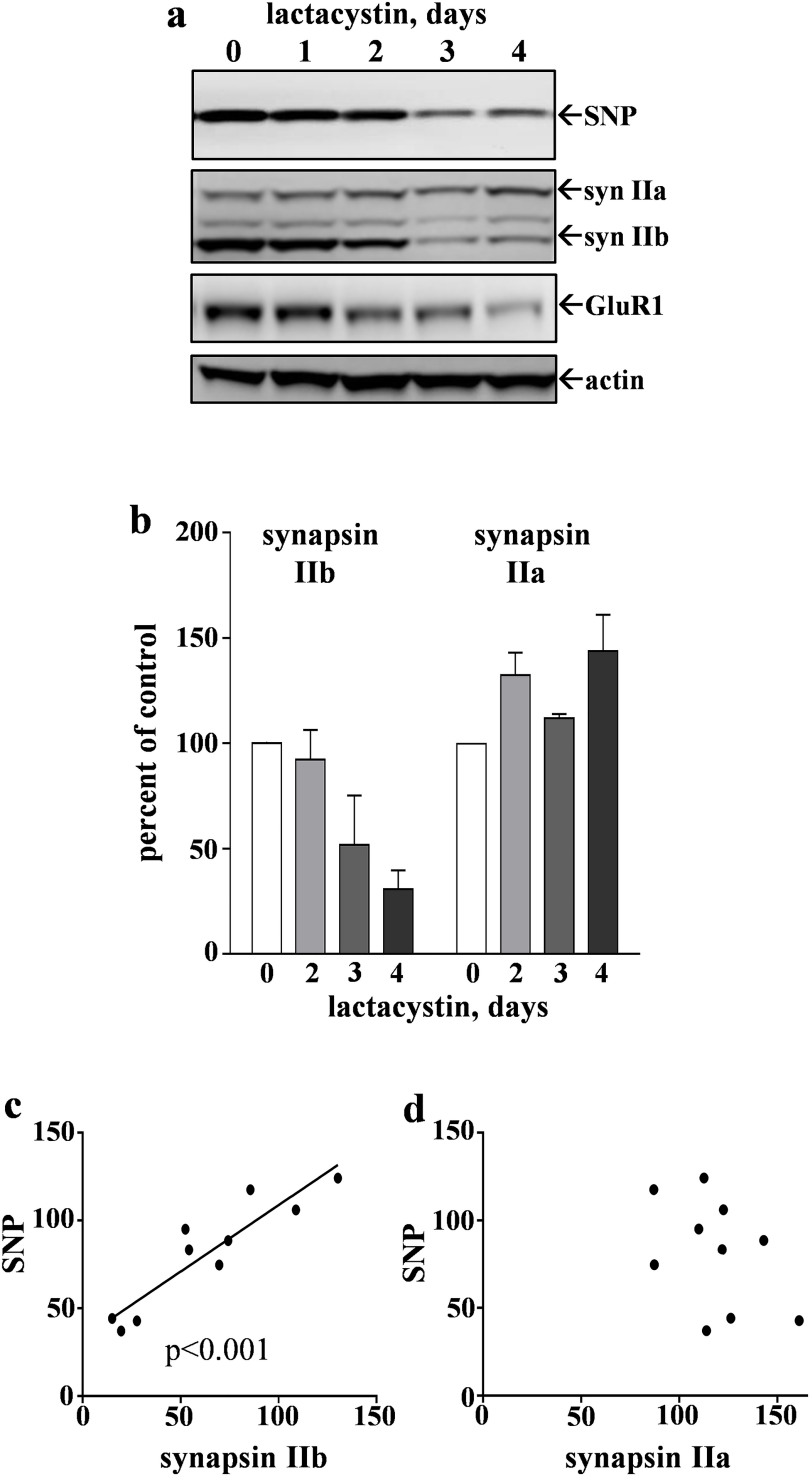
The proteasome inhibitor lactacystin causes delayed synaptic marker loss in hippocampal slice cultures. The hippocampal slices were treated daily with vehicle for 4 days (0-day control group) or with 5 μM lactacystin for 1–4 days, and treatments were staggered for same-day harvesting of 7–9 slices per group. The samples were assessed by immunoblot (a), staining the proteins synaptophysin (SNP), synapsin IIa (syn IIa) and IIb (syn IIb), GluR1, and actin. Mean synapsin levels were normalized to their respective controls and percent ± SEM values are shown for the time points with distinct effects between the two isoforms (b). Across the set of immunoblot samples, the levels of synaptophysin were plotted against the within-sample measures of synapsin IIb (c) and synapsin IIa (d). Linear regression analysis was conducted (c: R = 0.913, p<0.001; d: R = -0.430, N.S.).

The CatB response induced by lactacystin does not appear to correspond with the development of secondary pathology, in this case the delayed loss of synaptic proteins. Enhancement of CatB activity was occurring after 1–2 days of lactacystin treatment (Table 1), whereas the loss of synaptophysin and synapsin IIb in particular did not become evident until slices were treated for 3–4 days (Fig 6a). In the same lactacystin-treated samples tested for proteasome and CatB activities, 46–60% reductions at latter time points were evident by immunoblot staining for synaptophysin (p = 0.0015), GluR1 (p<0.01), and synapsin IIb (p<0.01), while no change in synapsin IIa was found (see Fig 6a and 6b). The loss of synapsin IIb is a distinct indicator of synaptic decline induced by lactacystin since Aβ_42_ had little to no effect on synapsin II isoforms (data not shown) as previously reported [50]. This more extensive effect on synaptic integrity may be due to lactacystin causing more proteasomal inhibition than Aβ_42_, noting that the residual proteasome activity (~50%) after 4 days of Aβ_42_ treatment was blocked to the level of 98.5% inhibition when lactacystin was included during the Aβ_42_ incubation. Also distinctive to lactacystin was the strong relationship between levels of the two susceptible markers, synapsin IIb and synaptophysin (Fig 6c; correlation coefficient of 0.913) across a set of treatment samples, whereas no relationship was evident between synapsin IIa and synaptophysin (Fig 6d).

**Fig 7:**
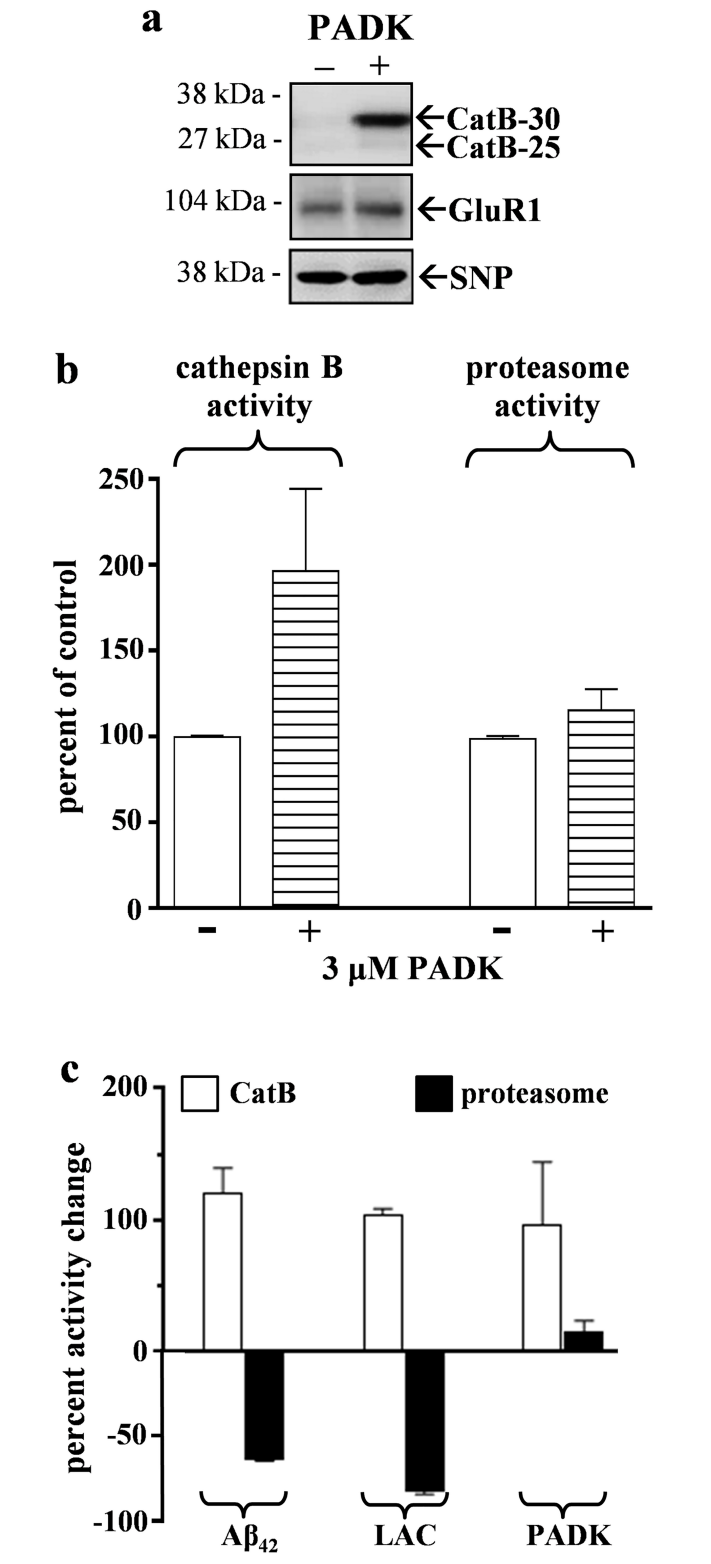
The effective CatB-enhancing agent PADK (chosen from Table 2) selectively enhances CatB activity in hippocampal slice cultures. The slice cultures were treated daily with vehicle or 3 μM PADK for 2 days before being collected into slice groups of 7–9 each. Panel a: Immunoblot assessments stained the 30- and 25-kDa CatB isoform (CatB-30 and CatB-25), GluR1, and synaptophysin (SNP). Panel b: Fluorogenic peptide assays assessed the harvested slice samples for cathepsin B and proteasome chymotrypsin-like activities. The two measures were normalized to their respective vehicle control groups (mean ± SEM). Cathepsin B activity exhibited a significant increase, whereas proteasome activity exhibited only a small increase. Panel c: Percent changes in CatB (white) and proteasome (black) activities compared to control are shown for 6-day Aβ_42_ treatment (from Fig 3a data), for 4-day lactacystin treatment (from Fig 4a and Table 1 data), and for 2-day PADK treatment (from Fig 7b data).

Since proteasomal inhibition by two different stressors, Aβ_42_ and lactacystin, led to increased CatB activity, we next tested whether enhancing CatB leads to reduced proteasome activity to follow the inverse relationships identified in the above experiments. From the screening of compounds that target CatB and related proteases (Table 2), those with very weak abilities to inhibit CatB curiously exhibited the ability to up-regulate levels of the 30-kDa active CatB isoform in hippocampal slices. Of note, modest dose-dependent increases were found with E64d (ANOVA: p<0.0001) and Cathepsin Inhibitor 1 (p = 0.0018). Z-Phe-Ala-diazomethylketone (PADK) at 1–10 μM caused a 3- to 6-fold selective increase in CatB-30 (ANOVA: p<0.0001) compared to unchanged synaptic markers (see Fig 7a) and the actin load control (not shown), as also previously reported for CatB activity in *in vitro* and *in vivo* studies [35, 47]. Thus PADK was chosen as a very effective CatB-enhancing agent to be used to test for inverse effects on the protein clearance pathways. When a set of hippocampal slice cultures were treated with 3 μM PADK and subsequently washed and assessed for both proteasomal and CatB proteolytic activities, a significant two-fold increase in CatB activity was found (Fig 7b, left bars) which was blocked if the PADK incubation included the potent CA074 inhibitor of CatB (not shown). With regards to proteasomal function, rather than a decrease for an inverse effect with respect to the CatB modulation, PADK elicited a 15% increase in proteasome activity, albeit not quite statistically significant (Fig 7b, right bars).

**Table 2:** Among screened compounds, PADK was chosen as an effective CatB-enhancing agent for further testing in the hippocampal slice model.

compound	CatB-30, percent ± SEM (n)	IC_50_ for CatB, μM
vehicle control	100.0 ± 4.0 (11)	–
PADK 1 μM	340.0 ± 38.7 (4)**	9–11 (from ref. [35])
3 μM	462.1 ± 95.8 (6)***	
10 μM	649.1 ± 74.5 (9)***	
SD1002 10 μM	345.0 ± 13.3 (4)**	>50 (from ref. [39])
E64d 1 μM	115.0 ± 9.2 (4)	14 (from ref. [53])
3 μM	122.2 ± 17.7 (3)	
10 μM	176.1 ± 12.5 (6)***	
E64 0.03–0.05 μM	144.0 ± 12.3 (4)*	0.03 (from ref. [54])
1–2 μM	111.5 ± 7.5 (4)	
Cathepsin Inhibitor-1 0.3–1 μM	120.9 ± 16.4 (3)	weak inhibitor (from ref. [55])
10 μM	149.4 ± 14.3 (3)**	
CA074 0.05 μM	89.0 ± 15.9 (3)	0.004 (from refs. [53, 54])
0.3–1 μM	95.3 ± 11.9 (3)	
CA074me 0.3–1 μM	116.0 ± 9.2 (6)	0.12 (from ref. [35])
lactacystin 5 μM	117.0 ± 20.3 (7)	>100 (from ref. [56])

The slice cultures were treated daily with vehicle or with the noted concentrations of selected compounds for 2–3 days. Tissue was then collected into slice groups of 7–9 each for immunoblot analysis. Detection of the 30-kDa CatB isoform (CatB-30) was measured in equal protein aliquots of samples and normalized to vehicle-treated control samples. Unpaired t test compared to vehicle treatment:

*p<0.05.

ANOVA multiple comparison tests compared to vehicle control:

**p<0.01.

***p<0.001.

A wide range of IC_50_ values for inhibiting CatB activity is provided for the ineffective to very effective compounds, obtained from the listed references.

**Fig 8:**
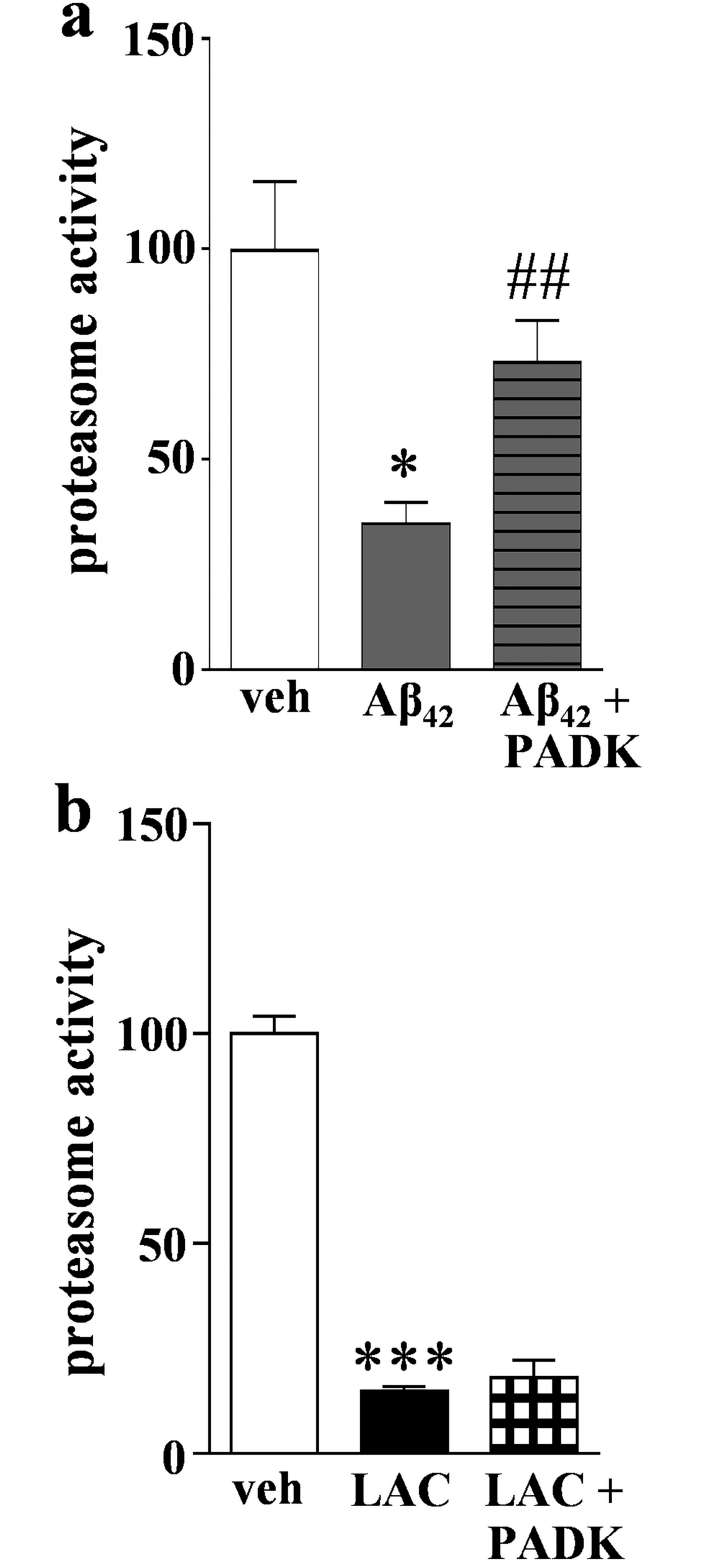
The CatB-enhancing compound PADK positively modulates the proteasome pathway in Aβ_42_–treated hippocampal slice cultures. Panel a: Slice cultures were treated daily with vehicle (veh) or 1.5 μM pre-aggregated Aβ_42_ for 6 days, or they were pre-treated with 3 μM PADK for 1 day before starting the 6 daily Aβ_42_ treatments in combination with 3 μM PADK. Proteasome activity was measured with a fluorogenic peptide assay and Vmax/s measures were normalized to vehicle-treated samples (mean ± SEM). Unpaired t test compared to control: *p = 0.0196; compared to Aβ_42_ alone: ##p = 0.027. Panel b: Slice cultures were treated with vehicle (veh), 10 μM lactacystin (LAC), or with 10 μM LAC in the presence of PADK for 2 days. Proteasome activity was assessed in harvested slices and measures were normalized to vehicle-treated samples (mean ± SEM). Unpaired t test compared to vehicle control: ***p<0.0001.

In contrast to the inverse effects that Aβ_42_ and lactacystin produced on the proteasomal and lysosomal pathways, the CatB positive modulator PADK did not produce such results since it clearly did not have a negative effect on proteasome function (Fig 7c). Surprisingly, PADK’s small enhancing effect on proteasome activity was much more pronounced in hippocampal slices that were treated for 6 days with Aβ_42_ peptide. The Aβ_42_–compromised proteasome activity (65% reduction) was improved more than two-fold by the co-application of PADK during the entire 6- day protocol (Fig 8a), reaching levels comparable to those in control slice cultures. From the statistical test, pre-aggregated Aβ_42_ was unable to produce a significant decline in proteasomal function in the presence of 3 μM PADK. It was also determined that PADK uniquely modulates the proteasome protein clearance pathway in Aβ_42_–treated brain tissue since the lactacystin-treated slices, those exhibiting more proteasomal inhibition than that produced by Aβ_42_, were unaffected by PADK with regards to recovery of proteasome function (Fig 8b).

**Fig 9:**
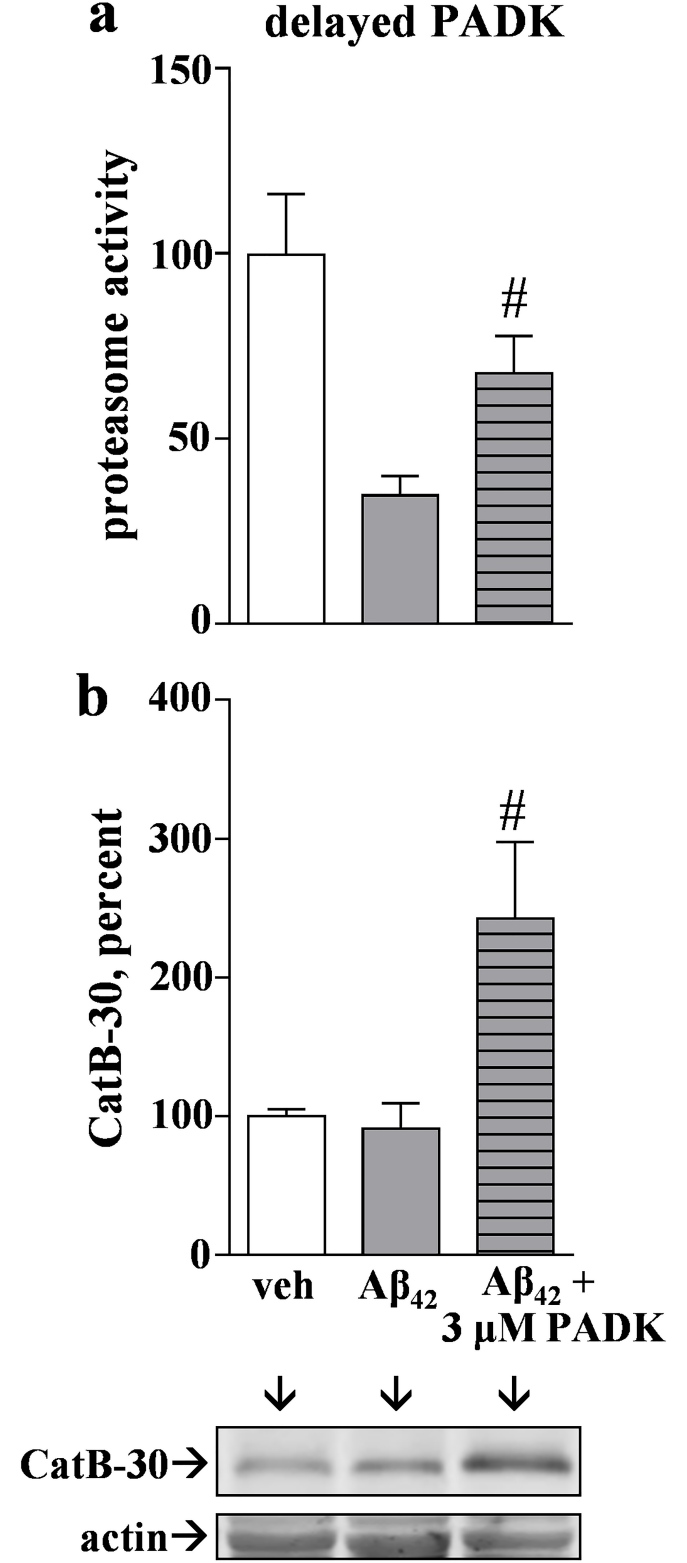
Aβ_42_–induced decline in proteasome activity is diminished by delayed PADK treatment. Panel a: Hippocampal slice cultures were treated daily with vehicle (veh) or 1.5 μM Aβ_42_ for 6 days, or they were subjected to the 6 daily Aβ_42_ treatments but 3 μM PADK was included during the last 3 days of Aβ_42_ incubations. Proteasomal activity used a fluorogenic peptide assay in harvested slice samples and Vmax/s measures were normalized to vehicle-treated control samples (mean ± SEM). Panel b: The harvested samples were also assessed by immunoblot for the 30-kDa active CatB isoform (CatB-30) and the actin load control. Mean CatB-30 levels were normalized to control values and percent ± SEM values are shown. Unpaired t tests compared to Aβ_42_ alone: #p<0.05.

Since pre-aggregated Aβ_42_ caused a delayed increase in CatB activity with no evidence of recovery regarding proteasome activity (see Fig 3a), we tested whether PADK can improve proteasome function when applied in a delayed manner, being administered after the first 3 days of the 6-day Aβ_42_ treatment protocol. As with continuous exposure, the delayed application of 3 μM PADK was still able to attenuate the Aβ_42_–induced decline in proteasome activity (Fig 9a). In association with the nearly two-fold improvement in proteasomal function, the active CatB-30 isoform was also up-regulated when daily inclusion of PADK was delayed 3 days (Fig 9b). Of particular note, the 6-day Aβ_42_ treatment alone did not affect CatB-30 levels. Thus, the up-regulation of CatB activity by both Aβ_42_ and lactacystin occurs independent of altered CatB-30 levels, indicating a distinct mechanistic difference compared to PADK’s robust increase in the active CatB isoform in association with activity modulation.

**Fig 10:**
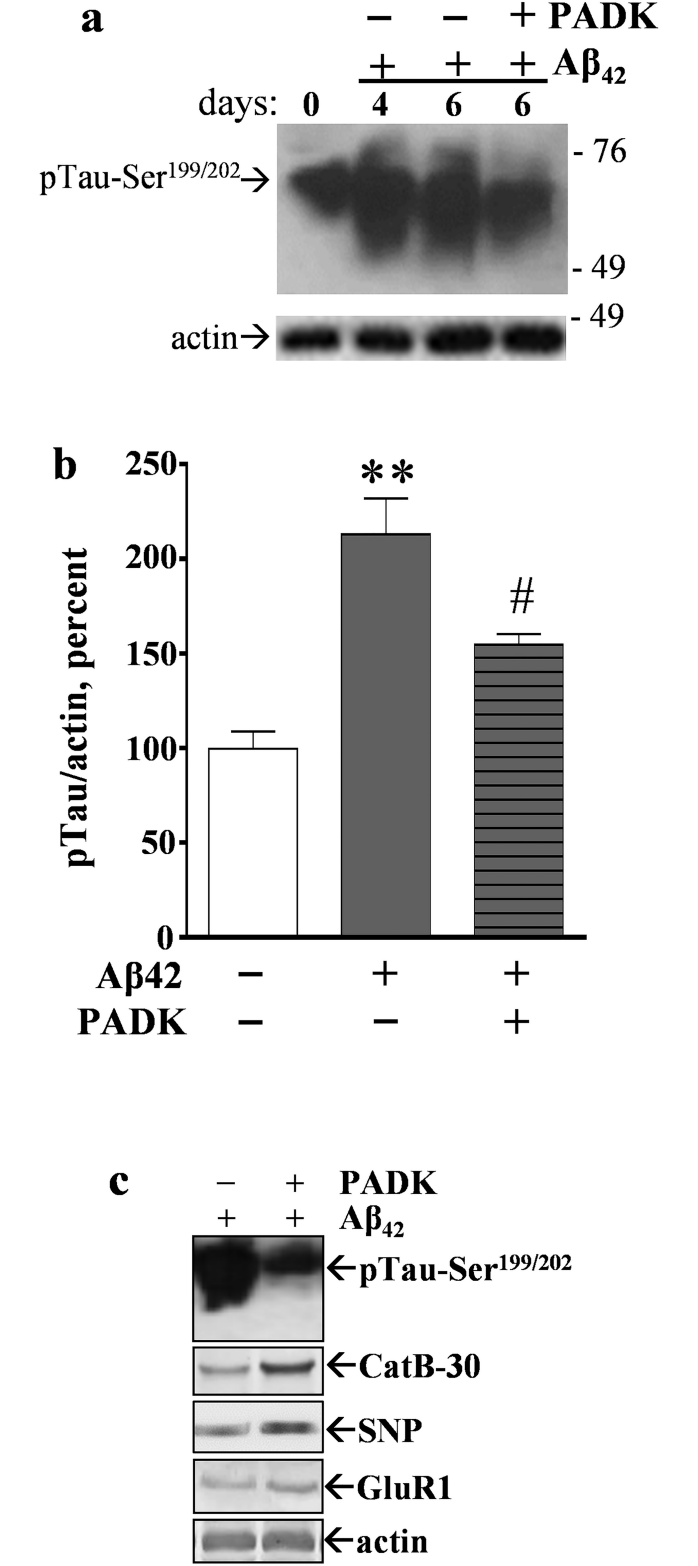
Aβ_42_–induced increase in phosphorylated tau is attenuated by PADK. Hippocampal slice cultures were treated daily with vehicle for 6 days (0-day control) or with 1.5 μM pre-aggregated Aβ_42_ for 4–6 days in the absence of presence of 3 μM PADK. The treatment schedule was staggered in order for same-day harvesting of 7–9 slices per group. Equal protein aliquots of the slice samples were assessed by immunoblot with antibodies against phospho-tau-Ser^199/202^ and against actin (a). Positions of molecular weight standards of 49–76 kDa are shown on the right. Integrated optical densities were measured and within-sample ratios between the two antigens were plotted for the 6-day treatments (b); note that the ratios were normalized to vehicle-treated samples (mean ± SEM). Unpaired t tests compared to vehicle control: **p<0.01; compared to Aβ_42_ alone: #p<0.05. Additional immunblot samples were stained for phospho-tau-Ser^199/202^, the 30-kDa CatB isoform (CatB-30), synaptophysin (SNP), GluR1, and actin (c).

**Fig 11:**
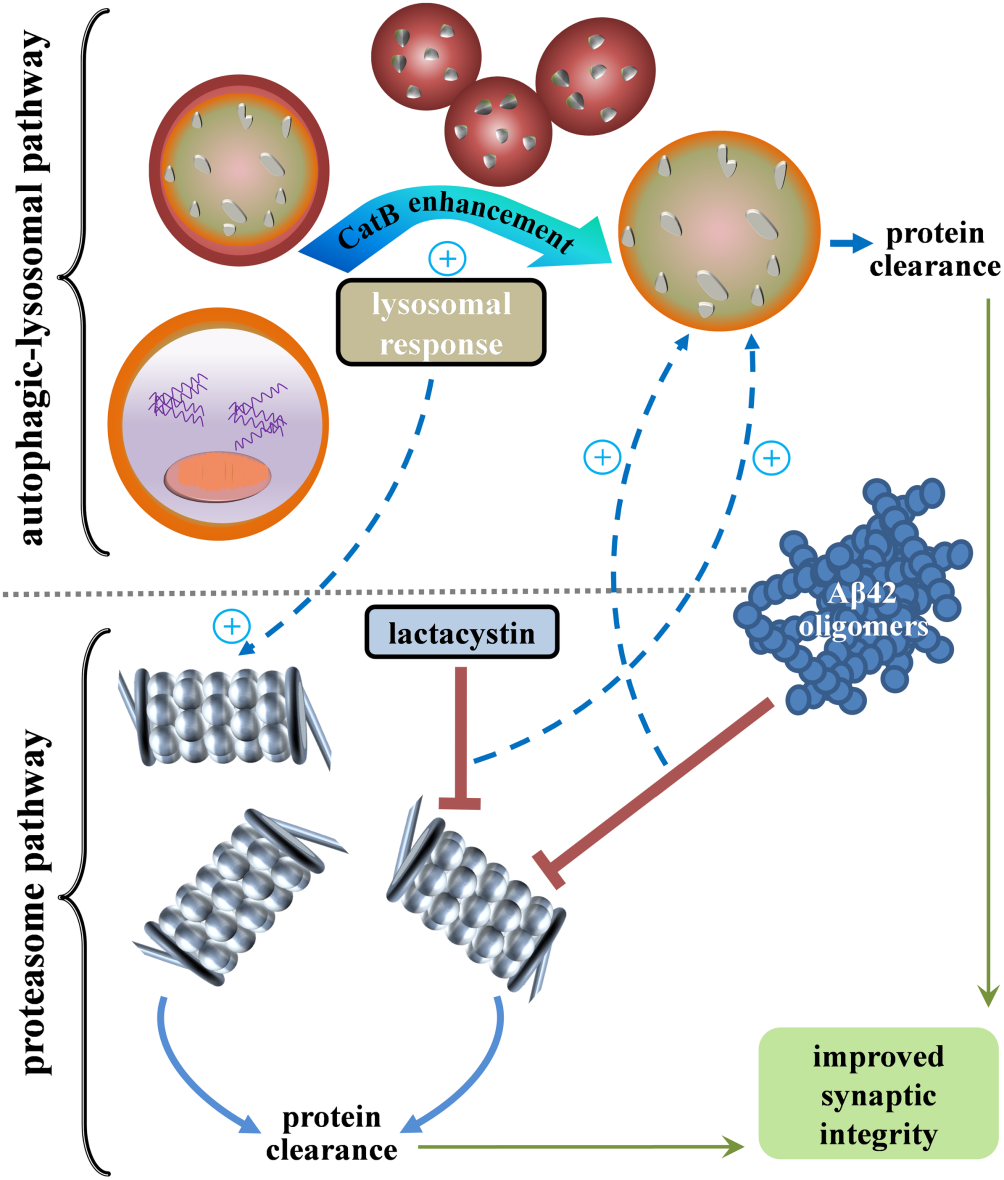
Model of the putative crosstalk between the two major protein clearance pathways. The autophagic-lysosomal system consists of autophagosomes, endosomes, primary lysosomes, and secondary lysosomes. The proteasomal system is composed of multimeric complexes with a catalytic core and regulatory subunits. Oligomerized Aβ_42_ and lactacystin elicit proteasomal inhibition which is linked to positive crosstalk with a component of the lysosomal pathway. Enhancing such compensatory lysosomal responses with a CatB-enhancing agent also leads to positive crosstalk back to the proteasome system.

In the Aβ_42_–treated hippocampal slice cultures, the PADK compound had a positive influence on both the proteasome system and the CatB enzyme of the autophagic-lysosomal system. Accordingly, the experimental samples were also tested for PADK effects on the indications of Aβ_42_–induced secondary pathology in the slice model. As shown in Fig 10a, PADK reduced tau phosphorylation mediated by the low-concentration Aβ_42_ incubations, perhaps due to multiple influences on protein clearance pathways. The hippocampal slices treated with pre-aggregated Aβ_42_ exhibited a two-fold increase in levels of phospho-tau-Ser^199/202^ and these levels were reduced by nearly 50% when PADK was present during the 6-day Aβ_42_ treatment period (Fig 10b). Additionally, the decrease in phospho-tau species corresponds with PADK’s enhancement of the active CatB isoform and improved levels of synaptic markers (Fig 10c). Hence, enhancement of the crosstalk that occurs in response to proteasomal stress appears to govern secondary events linked to Aβ_42_ pathology (see Fig 11).

## Discussion

In this study, low-level Aβ_42_ reduced proteasome activity in slice cultures from the hippocampus, a brain region vulnerable to AD, and the proteasomal compromise was associated with increased levels of phosphorylated tau species. These results indicate that AD-related proteasome dysfunction may be due to alterations in regulatory factors of the ubiquitin-proteasome system, leading to protein accumulation stress and perhaps neurofibrillary pathology. The important proteasome pathway has previously been shown to efficiently degrade misfolded and ubiquitinated proteins [57], and functional impairment of proteasomes impacts aging and age-related neurodegenerative diseases [1, 14, 13]. From the current results, proteasome dysfunction appears to tie together two key components of AD multi-proteinopathy, the Aβ_42_ peptide and tau, while leading to synaptic marker reductions in the slice model. The Aβ_42_ peptide was self-aggregated before being applied to the cultured brain tissue in order to resemble the oligomeric species present in AD brains. Note that Aβ_42_ oligomers, but not the monomeric form, were found to cause significant inhibition in a cell-free proteasome assay [16]. In the hippocampal slice model, the proteasomal protein clearance pathway is distinctly vulnerable to Aβ_42_ as compared to a lysosomal enzyme (CatB) that was not disrupted by the oligomerized peptide. Similar to Aβ_42_ treatment, disrupting the proteasomal pathway with the inhibitor lactacystin also led to secondary pathology in the hippocampal slices as indicated by the loss of synaptic proteins. Also common between Aβ_42_ and lactacystin, they both triggered a distinct type of lysosomal response involving the CatB enzyme.

The importance of protein clearance systems in AD is noted by the fact that Aβ peptides exhibit decreased clearing rates in the disease [44]. As suggested by the present results, early Aβ_42_ oligomerization may gradually disrupt proteasome function and thereby lead to reduced clearance of APP fragments, Aβ peptides, tau species, and perhaps other proteins involved in AD-related protein accumulation pathology. Aβ_42_-induced synaptic decline in the slice cultures may be partly an indirect effect mediated by proteasome inhibition since both Aβ_42_ and lactacystin caused significant reductions in synaptic proteins. Such loss of synaptic markers by low-concentration Aβ_42_ was found previously with regards to pre- and postsynaptic constituents found reduced in AD [50]. Additionally, lactacystin has been shown to induce another type of protein accumulation pathology in which the proteasome inhibitor was administered intranigrally for a mouse model of Parkinson’s disease [58]. The evidence linking a compromised proteasome system with synaptic pathology adds to studies showing that proteolysis through proteasomes is important for synaptic mechanisms and plasticity processes [51]. A series of steps may underlie the synaptopathogenesis [45], perhaps initiated by cellular internalization of the applied Aβ_42_ [59], the intraneuronal oligomers subsequently inducing tau pathology [60, 61], and altered tau species then interfering with proteasome function [14, 15]. It is feasible that the different protein accumulation events act synergistically on a variety of cellular mechanisms to disrupt synaptic integrity (e.g., through microtubule destabilization, impaired cellular transport, axonopathy, and/or ion channel deregulation).

Interestingly, the negative effect Aβ_42_ had on proteasomes was associated with a delayed enhancement of the lysosomal system in the form of an increase in CatB activity 6 days after initiating the Aβ_42_ treatment. When assessing the two protein clearance pathways after 4 days with Aβ_42_, the proteasome pathway appears to be selectively targeted since no change in CatB activity was evident in the slice cultures. The proteasome inhibition produced after 4–6 days of Aβ_42_ incubations was associated with synaptic marker loss and increased levels of phospho-tau. Noting that the up-regulation of CatB activity did not correspond with the initial elevation of pTau-Ser^199/202^ species, perhaps the delayed CatB response is only triggered by the subsequent higher levels of phospho-tau or by some interaction between the applied Aβ_42_ oligomers and the induced tau deposition that requires extra time to manifest. Note that human neuroblastoma cells treated with Aβ_42_ also exhibited proteasome inhibition and deregulation of the autophagic-lysosomal pathway, the latter confirmed by increased cathepsin activity [17]. In addition, treatment with Aβ_42_ but not Aβ_40_ was shown to increase both CatB mRNA and enzyme activity in the mouse N2A neuronal cell line [31]. Aβ_42_ levels 10–20 times higher than what was used here were found to elicit earlier cathepsin responses in slice and neuronal cultures [29, 31]. Thus, Aβ_42_ at a concentration more relevant to human AD points to proteasomal compromise as being associated with a delayed lysosomal response.

A positive CatB response was evident when proteasomes were disrupted by either Aβ_42_ or lactacystin in hippocampal slices, thus furthering the idea that the proteasomal and lysosomal systems are not independent protein clearance pathways. In both cases, the proteasomal dysfunction resulted in enhanced levels of lysosomal CatB activity. As previously noted, the two pathways appear to interact and the resulting crosstalk between proteasomes and the autophagy-lysosomal system may work to maintain protein homeostasis [12, 19, 21, 32]. The up-regulated CatB activity, induced by either Aβ_42_ or lactacystin, did not appear to correspond with secondary events of phospho-tau accumulation or synaptic marker reductions. Moreover, when CatB was enhanced with a selective modulator, no evidence of synaptic decline occurred when the compound was applied to hippocampal cultures alone, and it elicited protection of proteasome function and reduction in secondary pathology in the Aβ_42_–treated slice cultures. Note that the delayed CatB enhancement induced after 6 days of Aβ_42_ exposure may have in fact slowed the rate of tau changes since no further increase in phospho-tau levels was evident between 4 and 6 days of Aβ_42_ treatment. The PADK CatB-enhancing compound was more protective with regards to the tau alterations, perhaps due to its unique ability to greatly increase levels of the active CatB isoform. The modulation of CatB activity stemming from Aβ_42_– and lactacystin—induced proteasomal stress transpires independent of any evident changes in the CatB-30 isoform. The noted modulation events are indicative of positive crosstalk between the major protein clearance systems, as depicted in Fig 11, to potentially reduce protein accumulation stress and attenuate the pathogenic cascade that leads to synaptic dysfunction [45, 62]. Compensatory activation of a lysosomal enzyme may be in response to Aβ_42_ itself or through positive crosstalk stemming from the Aβ_42_–induced proteasomal stress.

Different types of protein accumulation stress have been shown to increase CatB levels in research models [29, 31]. Many questions obviously remain regarding CatB, particularly since the protease has been suggested to be a biomarker of AD as higher CatB protein levels were found in serum and plasma samples from persons with AD [63, 64]. However, recent work with human AD hippocampal samples did not show a correlation between CatB and the degree of AD pathology [65]. In their study, laser-captured CA1 pyramidal neurons from cases assigned a Braak stage III diagnosis exhibited a 35% increase in CatB protein as compared to nondemented control groups, whereas a smaller, insignificant change in CatB was found in neurons from the more severe Braak stage V. Thus, the reported higher CatB levels in AD may be an indication of a compensatory lysosomal response, and perhaps the weaker response in more severe cases explains an increased vulnerability that leads to worsening cognition and higher Braak rating. Note that compensatory responses are likely vital to maintain protein homeostasis and may act to slow protein accumulation pathology, perhaps explaining the slow nature of AD that tends to gradually worsen over 5–20 years in some individuals. In fact, indicators of protein accumulation stress in the AD brain include the induction of the autophagy-lysosomal pathway as well as impairment in lysosomal maturation [6, 7]. Also considering the compensatory lysosomal response, any depletion in such responses by a protein clearance pathway would lead to increased vulnerability to pathogenic protein accumulation. Two reports indicate that compensatory responses by lysosomes are diminished or absent in aged animals. In hAPP mice with amyloidogenic events, CatB activity was increased in the hippocampus of young and middle-aged animals, but not in the elderly hAPP mice [31]. In a rat model of proteasome stress, compensatory crosstalk between the proteasome system and the autophagy-lysosome pathway was found in young but not aged rats, and restoration of protein homeostasis occurred only in hippocampal neurons of the young animals [32]. Thus, age-related changes in lysosomal compensatory responses may explain why age is a major risk factor of AD.

In regards to plasma CatB levels leading some to speculate a link to AD cognitive pathology, a more recent study found the opposite in monkeys and humans in which treadmill exercise elevated CatB in plasma and, moreover, the resulting CatB enhancement correlated with improved hippocampal-dependent memory [66]. These findings are consistent with animal models and human research indicating that physical activity can significantly offset the progressive loss of hippocampal function and prevent or delay the onset of age-related disorders like AD and AD-related dementias [67].

The CatB-enhancing PADK compound is a very weak cathepsin B and L inhibitor that augments the active mature form of CatB when applied to the hippocampal cultures alone. In regards to protein accumulation stress in the slice model, the PADK treatment reduced the Aβ_42_–mediated phospho-tau accumulation and associated synaptic decline. If the CatB up-regulation in response to Aβ_42_–induced proteasomal stress is part of a compensatory feedback pathway, then it stands to reason that a compound that promotes the compensating signal would further protect against secondary pathology linked to the disrupted proteasomes. It is noteworthy that other weak cathepsin inhibitors were also found to up-regulate active CatB including *i*) SD1002, a nonpeptidic PADK analogue previously shown to promote Aβ_42_ clearance and synaptic integrity in AD mice [39], *ii*) Cathepsin Inhibitor-1, a broad inhibitor of papain and cathepsins B, G, L, and S, and *iii*) E64d, a membrane-permeable inhibitor of several cysteine proteases that has been reported to inhibit CatB to reduce Aβ and improve memory in APP mice [42]. However, studies also indicate that the latter compound blocks both the calcium-activated protease calpain and CatB to protect against other neuropathogenic insults [53, 68] and can elicit neuroprotection through a CatB-independent pathway [69]. The positive response through CatB may be an influential factor in AD pathogenesis, allowing the lysosomal system to play an important role in governing Aβ pathology. While there is conflicting evidence regarding CatB in the development of AD, it should be pointed out that *i*) CatB was found to cleave Aβ_42_ into less amyloidogenic species [31], *ii*) the C-terminally truncated products Aβ_38_ and Aβ_33_ appear to be selectively cleaved from Aβ peptides by purified CatB [31, 34], and *iii*) production of the Aβ_38_ product corresponds with improved Aβ_42_ clearance in transgenic mice treated with the CatB-enhancing compound PADK [35]. Aβ peptides were also reduced by genetic and pharmacological avenues that increase CatB activity in mice expressing hAPP with AD-linked mutations [31, 35, 37–39, 41, 48] or wild-type hAPP [40], as well as in neural progenitor cell-derived neurons expressing mutant hAPP [41]. As mentioned, induction of the autophagy-lysosomal pathway has long been suggested as being neuroprotective against protein accumulation disorders. The very weak inhibitors appear to promote CatB maturation and may provide dual modulation of the separate systems for clearance of pathogenic proteins.

The results in this study suggest that AD-related proteasomal stress contributes to distinct protein accumulation pathology. Age-related protein accumulation events may create circumstances that strain the proteasomal system, leaving the brain more vulnerable to AD-type pathogenesis. If proteasome function is in fact vulnerable to the early buildup of Aβ_42_ in AD, protective modulation of the proteasomal system would be a viable treatment strategy. It is of interest that the lysosomal system responds to proteasome inhibition and that a CatB-enhancing agent elicits recovery of proteasomal function. Note that the autophagy-lysosomal system has long been considered a major factor for neuronal health and that induction of this lysosome-mediated degradative pathway is often found to be protective in animal models of neurodegenerative diseases [18, 19, 22, 24, 70]. The current findings further support the idea that interactions occur between the proteasome system and the lysosomal protein clearance system during episodes of protein accumulation stress. Together, the two pathways make up a complimentary network for cell homeostasis, and modulated balancing between the two pathways through compensatory responses is a likely cellular attribute for maintaining consistent clearing of old and mis-folded proteins.
